# Rhamnose Links Moonlighting Proteins to Membrane Phospholipid in Mycoplasmas

**DOI:** 10.1371/journal.pone.0162505

**Published:** 2016-09-07

**Authors:** James M. Daubenspeck, Runhua Liu, Kevin Dybvig

**Affiliations:** Department of Genetics, University of Alabama at Birmingham, Birmingham, Alabama, 35294, United States of America; Miami University, UNITED STATES

## Abstract

Many proteins that have a primary function as a cytoplasmic protein are known to have the ability to moonlight on the surface of nearly all organisms. An example is the glycolytic enzyme enolase, which can be found on the surface of many types of cells from bacteria to human. Surface enolase is not enzymatic because it is monomeric and oligomerization is required for glycolytic activity. It can bind various molecules and activate plasminogen. Enolase lacks a signal peptide and the mechanism by which it attaches to the surface is unknown. We found that treatment of whole cells of the murine pathogen *Mycoplasma pulmonis* with phospholipase D released enolase and other common moonlighting proteins. Glycostaining suggested that the released proteins were glycosylated. Cytoplasmic and membrane-bound enolase was isolated by immunoprecipitation. No post-translational modification was detected on cytoplasmic enolase, but membrane enolase was associated with lipid, phosphate and rhamnose. Treatment with phospholipase released the lipid and phosphate from enolase but not the rhamnose. The site of rhamnosylation was identified as a glutamine residue near the C-terminus of the protein. Rhamnose has been found in all species of mycoplasma examined but its function was previously unknown. Mycoplasmas are small bacteria with have no peptidoglycan, and rhamnose in these organisms is also not associated with polysaccharide. We suggest that rhamnose has a central role in anchoring proteins to the membrane by linkage to phospholipid, which may be a general mechanism for the membrane association of moonlighting proteins in mycoplasmas and perhaps other bacteria.

## Introduction

The genus *Mycoplasma* is composed of minimalist wall-less bacteria that are obligate parasites. *Mycoplasma genitalium* has the distinction of having the smallest genome known for a free-living eubacteria, 470 predicted coding regions [1]. One consequence of this minimalism is host specificity and dependency. Mycoplasmas are dependent on their host for cholesterol and lipids. Despite such a limited genome, this genus synthesizes polysaccharides, glycosylates proteins, forms biofilms, and has a multi-component, sophisticated host immune avoidance system [2,3]. It also has all the systems required for a free-living, self-replicating bacterium. It is difficult to rationalize the complexity of the organisms with their limited proteome. One potential mechanism for expanding the functionality of the proteome is for some proteins to have multiple functions or to “moonlight”.

Moonlighting proteins are ubiquitous. In every possible category of life, from bacteria to humans, investigators have found critical and essential cytoplasmic enzymes on the surface of the cell, involved in functions that are far removed from their primary purpose. The murine pathogen *Mycoplasma pulmonis* was chosen for the study of moonlighting proteins as more is known about the glycosylation of its proteins as compared to other mycoplasmas [2,4–6]. We chose to focus on moonlighting molecules of phosphopyruvate hydratase (enolase), due to its abundance and the commercial availability of reagents. Enolase is a cytoplasmic multimeric metalloenzyme that converts 2-phosphoglycerate to phosphoenolpyruvate, the ninth and penultimate step in glycolysis. This enzyme is highly conserved across species—from mycoplasma to humans multimeric enolase is a critical component of the glycolytic cycle. In the same diverse group of species, monomeric enolase is found on the surface of the cell and has been shown to bind plasminogen, fibronectin, and laminin (http://www.moonlightingproteins.org) [7].

Cell surface enolase is important. Molecules of enolase that moonlight on the surface of bacterial and mammalian cells bind and activate plasminogen [8–10]. In some organisms, moonlighting molecules of enolase have also been shown to bind fibronectin, laminin, and the mucin MUC7 [7]. Wild-type enolase, and also a recombinant enolase that was mutated to be catalytically inactive, has a role in vacuole membrane fusion and the trafficking of protein to the vacuole in yeast [11]. Enolase is thought to have a role in adhesion in most species of mycoplasma [12–14] and other bacteria [15]. Enolase on the surface of the *Plasmodium* malaria agent has a role in tissue invasion [16,17]. In addition to these infectious agents, alpha-enolase on the surface of human cells has been implicated in the progression of Alzheimer’s disease [18], autoimmune disorders [19–21], and cancer invasion and metastasis [22].

A recent proteomics analysis of proteins on the surface of bacterial cells found more than 1000 out of 3619 proteins examined lacked the transmembrane domains or traditional signal peptide sequences associated with membrane-bound proteins [23]. It is unknown how these proteins are attached to the membrane. In the current study, we offer evidence for a mechanism of attachment and propose a model for the post-translational modification (PTM) responsible. Based on gas chromatography (GC), high-resolution mass spectrometry (HR-MS), and SDS-PAGE analysis, enolase is attached to the membrane through a rhamnophospholipid anchor. As rhamnose is widespread in the genus *Mycoplasma* with no previously identified function, it is likely that many proteins in this genus are attached to the surface by covalent attachment to phospholipid via a rhamnose linker [5].

## Materials and Methods

### Phospholipase D treatment

For all experiments, *M*. *pulmonis* strain CTG [2] was grown in DMEM (Cellgro) supplemented with 15% whole horse serum as described [4]. Cells from mid-log phase cultures were harvested by centrifugation and washed twice in PBS. The bacteria were suspended in 1 ml of 50 mM Tris and 50 mM CaCl_2_ at pH 8.0 and split into separate 500-μl samples. One sample was treated with 200 units of phospholipase D from *Streptomyces chromofuscus* (Sigma). The other sample was treated with the same concentration of heat-killed (10 minutes at 100°C) enzyme as a control. The reactions were carried out at room temperature for 90 minutes, with constant turning. The samples were dialyzed against 5000 volumes water to remove salts and analyzed by SDS-PAGE.

### Staining of SDS-PAGE gels

Proteins were analyzed on 4–15% gradient SDS-PAGE gels. Glycosylation was monitored using Pro-Q® Emerald 300, a periodic acid based stain (ThermoFisher). Phosphorylation was monitored utilizing Pro-Q® Diamond (ThermoFisher). Coomassie staining was done with standard laboratory reagents and standard techniques.

### Membrane purification

Bacteria washed twice in PBS were incubated overnight in 0.5 ml of 2 x PBS at 4°C. The cold samples were injected into 50 ml of H_2_O at 37°C to induce osmotic lysis. The material was centrifuged at 5000 x *g* for 10 minutes to remove whole cells and insoluble material. The supernatant was centrifuged at 36,000 x *g* for 60 minutes to pellet the membranes. The membrane pellet and the cytoplasmic supernatant were collected and analyzed as described. This is an adaptation of a protocol described by Razin [24].

### Immunoprecipitation (IP)

The proteins from the *M*. *pulmonis* membrane or cytoplasmic fractions were precipitated using chloroform and methanol [25]. The precipitated proteins were solubilized in 1 ml of 50 mM Tris at pH 8.0. The sample was incubated with the primary antibody overnight at 4°C. The antibody was to human enolase (polyclonal H-300 from Santa Cruz Biotechnologies). The sample was added to Separose 4B beads covalently linked to Protein A (Invitrogen) that had been preincubated with 5% BSA (overnight at 4°C). The sample was allowed to bind for 2 hours at 4°C. The bound material was washed twice in PBS, solubilized in SDS-PAGE non-reducing loading buffer, heated to 100°C for 10 minutes, and analyzed by SDS-PAGE.

### HR-MS

Protein bands containing enolase were excised and digested with trypsin. The tryptic peptides were subjected to LC-HR-MS using the methods as described [6]. The mass spectra were analyzed utilizing PEAKS 7 proteomics software (Bioinformatics Solutions, Inc.) to identify candidate spectra of a peptide containing a possible PTM. These spectra were analyzed in depth as described [4].

## Results

### Phospholipase D treatment of whole cells

**Fig 1:**
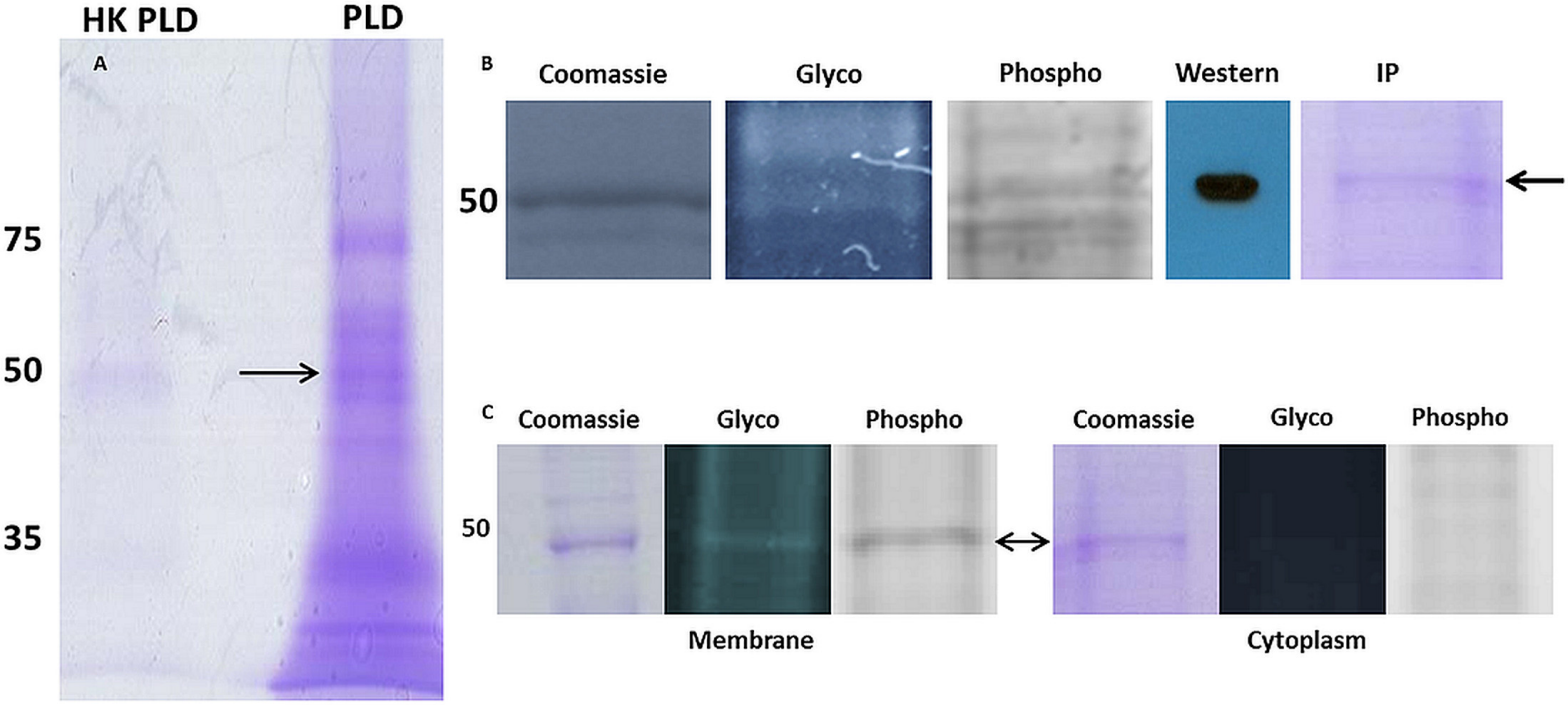
SDS-PAGE analysis of *M*. *pulmonis*. Panel A, Coomassie-stained gel of supernatants obtained by treatment of whole cells with phospholipase D (PLD) or heat-killed enzyme (HK-PLD). Proteins were eluted from the gel in wide swaths that were analyzed by ESI-TOF MS for protein identification (see Table 1). Panel B, membrane proteins were analyzed on gels stained with Coomassie, Pro-Q Emerald (Glycostain), Pro-Q Diamond (Phosphostain), Western blot reacted with antibody to enolase, and a Coomassie-stained gel of IP-purified enolase. Panel C, IP-purified enolase from membrane and cytoplasmic fractions were analyzed on gels stained for total protein (Coomassie), glycoprotein, or phosphoprotein. In all panels numbers refer to the masses of protein standards, and arrows refer to the expected location of enolase.

Phospholipase D (lipophosphodiesterase) hydrolyzes its primary substrate of phosphatidylcholine into phosphatidic acid and choline. We treated whole *M*. *pulmonis* cells with phospholipase D or heat-killed enzyme as a control. After one hour at room temperature, cells were removed by centrifugation and the supernatant analyzed by SDS-PAGE (Fig 1A). CFU analysis demonstrated that phospholipase D treatment had no effect on cell viability. A significant number of proteins were released from the surface by phospholipase D. Wide swaths of the phospholipase D-treated lane were digested with trypsin and analyzed by electrospray ionization-MS for protein identification (Fig 1A). The major proteins identified are listed in Table 1. Many of these proteins (MYPU_2230, 4050, 5180, 7300, 7610, and 7620) have orthologs that are known moonlighting proteins residing on the cell surface of various organisms (see the MoonProt database at http://www.moonlightingproteins.org). The mechanism by which these moonlighting proteins are associated with the membrane is generally unknown for any organism. Enolase (MYPU_5180) was selected as a model protein to study non-traditional membrane association because of its high abundance and the commercial availability of specific antibody.

**Table 1:** Proteins identified in supernatants after treatment with phospholipase.

Designation	Protein	References for surface localization in mycoplasma or other organisms
MYPU_2230	Chaperone DnaK*	[26,27]
MYPU_3210	Oligoendopeptidase F	
MYPU_3460	ABC transporter	
MYPU_4050	Elongation factor EF-Tu *	[28,29]
MYPU_4380	Similar to IgG-binding protein MIB (Protein M superfamily)	[30,31]
MYPU_5050	Similar to IgG protease MIP (Protein M superfamily)	[31]
MYPU_5180	Enolase *	[13,32,33]
MYPU_5280	Variable surface antigen VSA	[34,35]
MYPU_6510	Uncharacterized protein	
MYPU_7300	Glutamyl aminopeptidase *	[36]
MYPU_7610	Dihydrolipoyl dehydrogenase*	[28]
MYPU_7620	Dihydrolipoamide acetyltransferase*	[28]

*Indicates homologs identified as moonlighting proteins in mycoplasmas or other organisms.

### Staining and purification of enolase

Proteins from a membrane fraction of *M*. *pulmonis* were analyzed on SDS-PAGE gels stained with Coomassie, Pro-Q® Emerald 300 (glycoprotein stain), and Pro-Q® Diamond (phosphoprotein stain) (Fig 1B). Enolase was one of a number of proteins that appeared to react with all three stains. Enolase from *M*. *pulmonis* and the first 300 amino acids of *Homo sapiens* alpha enolase share 46% amino acid identity. We found that a rabbit polyclonal antibody against amino acids 1–300 of human alpha enolase reacted with enolase from *M*. *pulmonis* (Fig 1B). This antibody was used to isolate enolase from *M*. *pulmonis* by IP (Fig 1B).

Enolase from membrane and cytoplasmic fractions was isolated by IP. Enolase from the membrane fraction reacted with both the glycostain and the phosphostain (Fig 1C). Cytoplasmic enolase stained with Coomassie but exhibited no indication of glycosylation or phosphorylation. It was apparent that membrane-bound enolase has a PTM that is absent on the enzymatically active cytoplasmic enolase.

### GC/MS of IP-purified enolase

**Fig 2:**
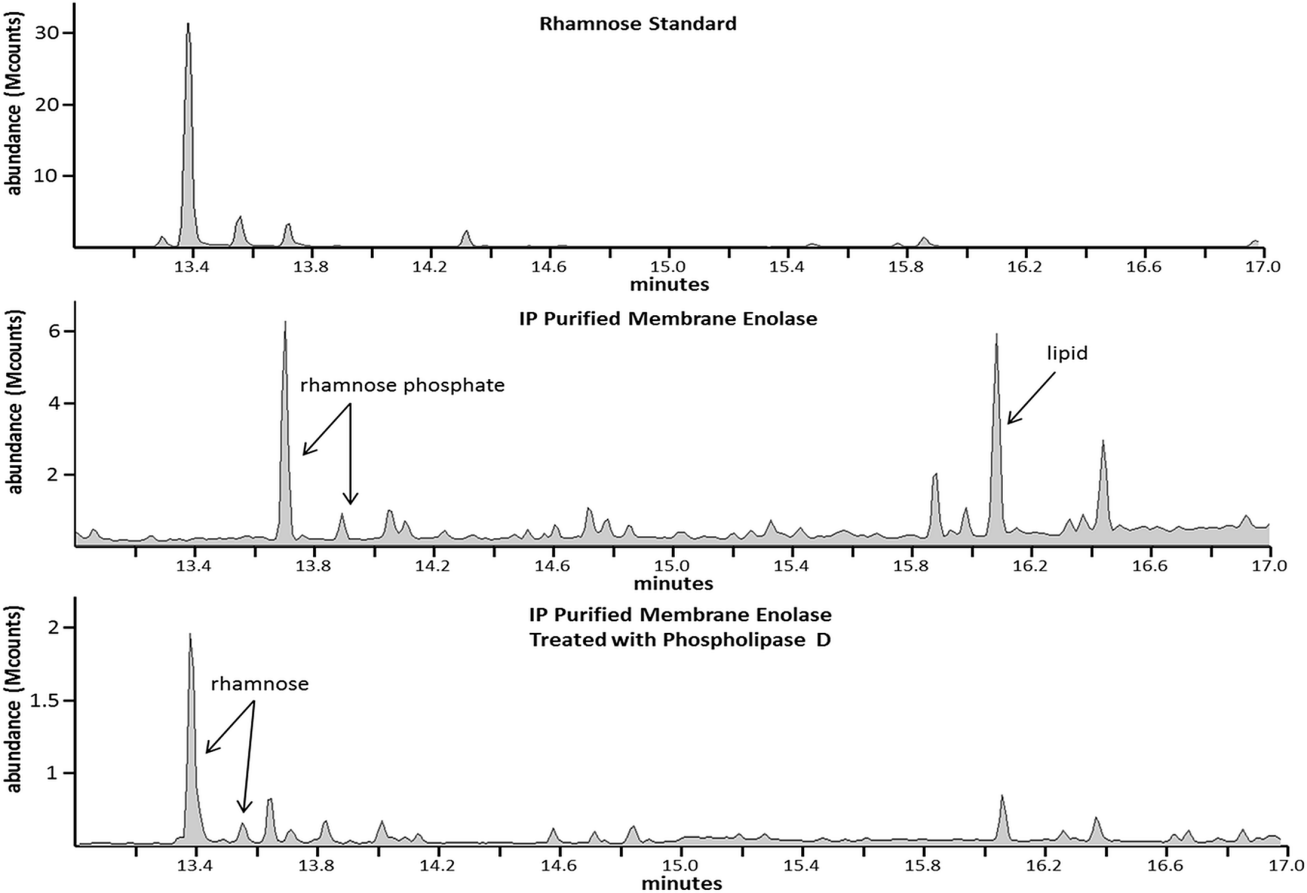
GC/MS of a rhamnose standard (panel A), IP-purified membrane enolase isolated from a SDS-PAGE gel (panel B), and the same material as analyzed in panel B after subsequent treatment with phospholipase D (panel C).

Membrane and cytoplasmic IP-purified enolase were digested with trypsin, extracted from a gel, and subjected to methanolysis to generate the methyl glycosides for analysis by GC. The cytoplasmic enolase produced a spectrum that failed to show any indication of a PTM, consistent with the results shown in Fig 1C. Fig 2 compares the chromatogram of the rhamnose standard (panel A) to that of membrane enolase (panel B). There are 2 peaks of interest in panel B. The first is what we have concluded to be a rhamnose phosphate and the second is a lipid. The assignment of the lipid peak is straightforward as the MS/MS clearly shows a repeating pattern of ions separated by 14 Da (S1 Fig), indicative of the fragmentation of a long chain fatty acid.

Methanolysis will cleave the sugar-phosphate bond at the anomeric or C1 position but phosphate linked at the other positions retains its association with the sugar [37,38]. The MS/MS of the rhamnose phosphate peak shown in Fig 2B is provided in S2 Fig. Panel A of S2 Fig shows the MS/MS of the rhamnose standard. The MS/MS of rhamnose is identical to that of other pyranoses, showing the ions 133, 147, 204 and 217 m/*z* that correspond to known fragments generated by derivatization by trimethylchlorosilane (TMS) (S2 Fig, panel B) [39,40]. The MS/MS spectra of the putative rhamnose phosphate (S2 Fig, panel C) shows these characteristic ions and additional ions of 116, 180, and 296 m/*z*. The ions at m/*z* 116 and 180 have been shown to be characteristic of a sugar phosphate [41]. The ion at m/*z* 296 is likely specific for rhamnose phosphate. Derivatization of glucose phosphate yields a major peak at 299 m/*z* [41]. The 296-m/*z* peak seen in panel C might result from the replacement of the oxygen atom of glucose with the CH_3_ group of the methylpentose (rhamnose) (S2 Fig, panel D). Panel C has another peak at 387 m/*z*, which has been shown to result from trimethylsilation of inorganic phosphate[41].

The data shown in Fig 2, panel B suggests that rhamnose is associated with phospholipid. To test this prediction, IP-purified membrane enolase was treated with phospholipase D and analyzed by GC/MS. Rhamnose was associated with membrane enolase after phospholipase treatment but no rhamnose phosphate or lipid was detected (Fig 2, panel C). These results indicated that the enzyme cleaved the phospholipid-rhamnose bond of membrane enolase.

### Analysis of enolase by HR-MS to identify PTMs

**Fig 3:**
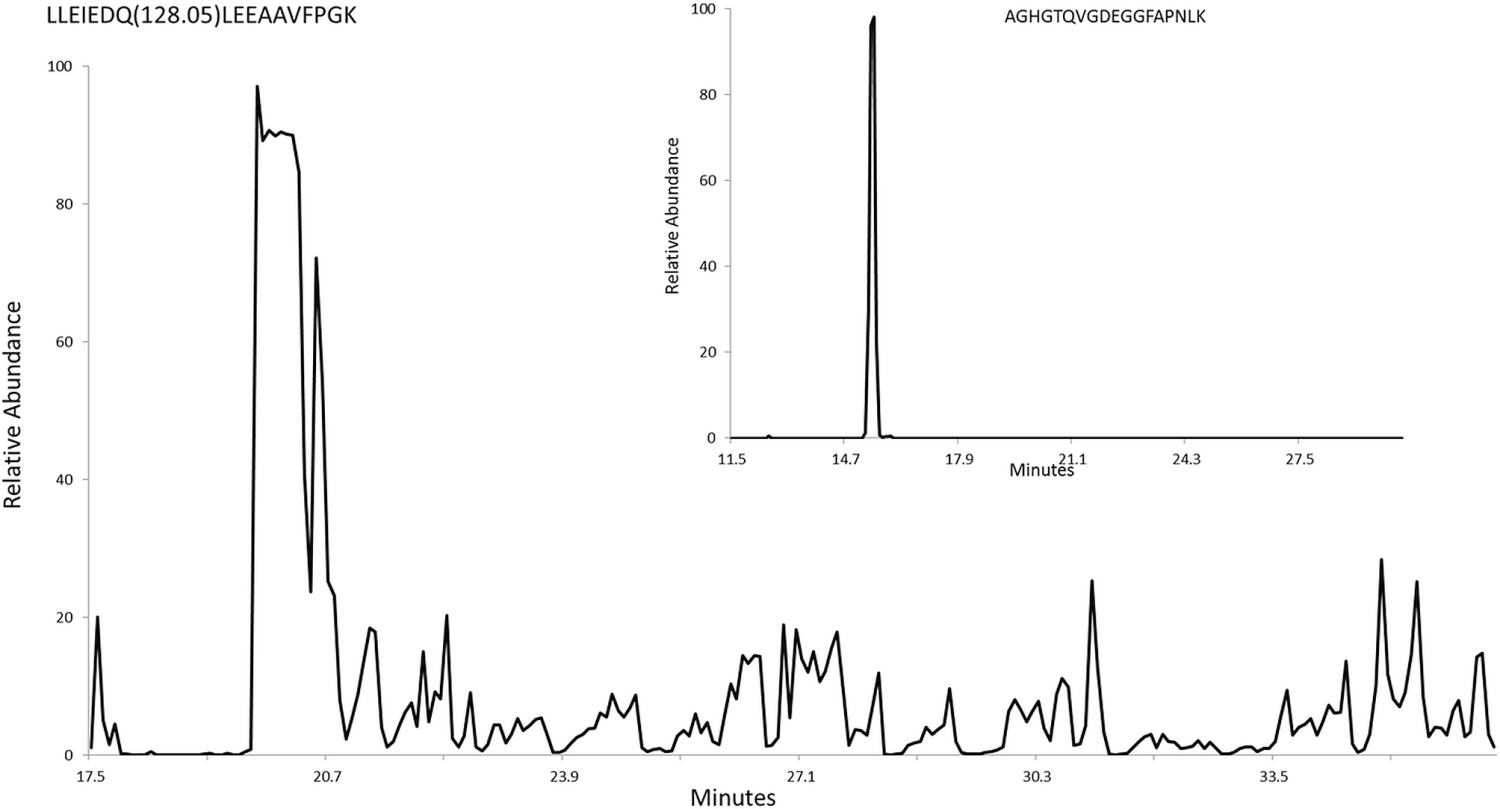
HPLC plot of the relative abundance of the LLEIEDQLEEAAVFPGK peptide of enolase. The inset is a plot of another peptide from enolase that lacks lipid modification.

**Fig 4:**
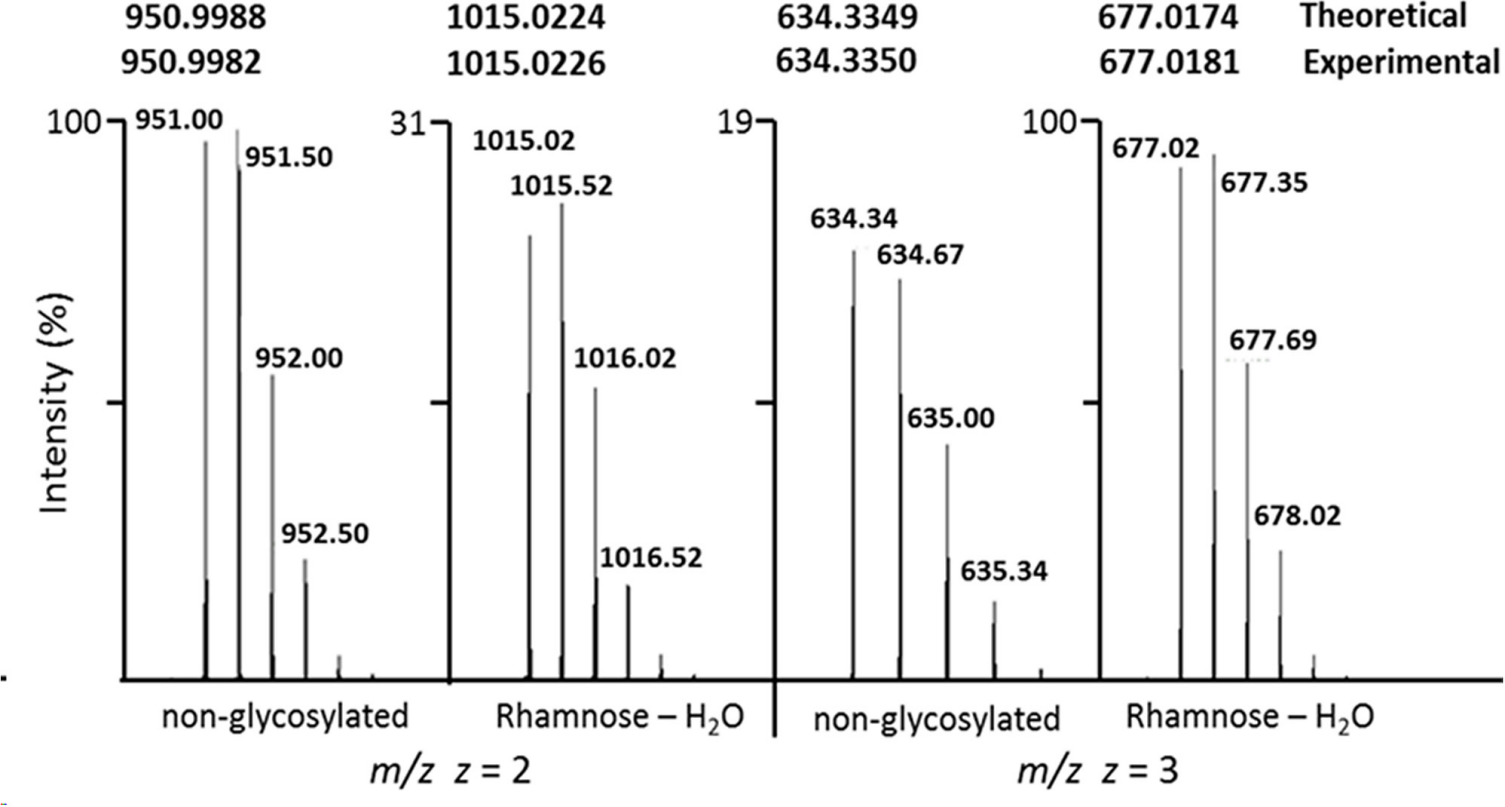
HR-MS of the LLEIEDQLEEAAVFPGK peptide showing the modified and unmodified states for *z* = 2 and *z* = 3. Numbers at the top are the theoretical and experimentally observed masses of the peptide.

**Fig 5:**
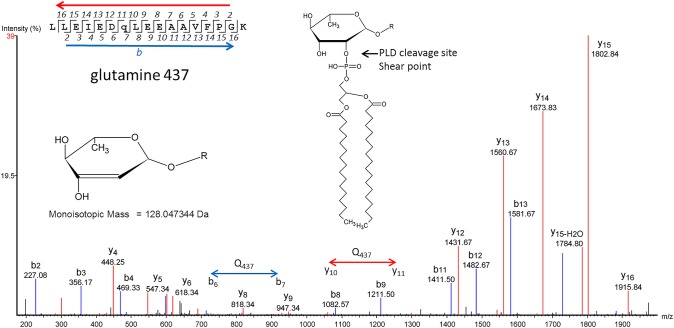
MS/MS of the LLEIEDQLEEAAVFPGK peptide showing modification at Gln437. Inset A. Model for the 128.05 m/*z* residue. Inset B. Model of the PTM associated with membrane enolase. The model is based on phosphatidylcholine because this lipid is a main substrate for phospholipase D and is a major lipid for mycoplasmas grown in serum [42].

Gel-purified enolase was analyzed by HR-MS using reverse phase HPLC. Tryptic peptides covering 90% of the protein were identified. Most peptides eluted from the HPLC column as a tight peak (Fig 3, inset). No glycosylation or phosphorylation was detected on any of these peptides. One peptide, LLEIEDQLEEAAVFPGK, did not purify as a tight peak and eluted from the C18 column throughout the run, suggesting the presence of a lipid moiety that would bind tightly to the column (Fig 3 and S3 Fig). It would be expected that proteins covalently linked to lipid would elute from the column at random times as a result of shearing, with the lipid component remaining on the column and the peptide released. This sheared peptide had evidence of a PTM of mass 128.05 Da. We identified both the modified and unmodified forms of the peptide in multiple charge states (*z* = 2 and *z* = 3), showing the modified form has a mass shift of 128.0473 Da. This mass corresponds exactly with the mass of rhamnose (146.0579 Da) minus one molecule (18.0106 Da) of HOH with a degree of accuracy of 0.0002 Da for *z* = 2 and 0.0007 Da for *z* = 3 (Fig 4). Shearing of the phosphoester bond between the phospholipid and rhamnose might be expected to result in loss of HOH (Fig 5 inset).

Previously, we have shown that mycoplasmas glycosylate serine, threonine, asparagine and glutamine[4]. The peptide containing the 128.05 modification has a single glutamine residue but lacks serine, threonine and asparagine. A liquid chromatography (LC) MS/MS-CID spectrum identified the 128.05 modification at glutamine (Gln437) (Fig 5). The mass for the assigned b_7_ ion is 128 Da greater than would be expected for the unmodified form. Similarly, the mass of the assigned y_11_ ion has a mass shift of 128 Da. In both cases, the mass shift occurs at Gln437. The assigned peaks for the spectrum are given in S1 Table.

## Discussion

Rhamnose has been identified in all species of mycoplasma examined but no function has previously been described [5]. We find here that rhamnose serves to link proteins of *M*. *pulmonis* to phospholipid. It is likely that this function is conserved throughout the genus *Mycoplasma*. Phospholipase D treatment of whole cells released a plethora of proteins from the cell surface including a number of known moonlighting proteins. In some cases, a protein might lack rhamnose and lipid but be released from the cells by phospholipase treatment because the protein was complexed to other proteins that were attached to phospholipid and released. Mycoplasmas possess about 8 ng rhamnose per ug of protein, but based on GC analysis we estimate that the level of rhamnose phosphate is only 40 pg per ug protein [5]. Assuming only 1 rhamnose-phosphate modification per protein, this level of rhamnose phosphate would be sufficient to modify about 8% of the protein content of the cell. We predict that dozens of proteins, not just enolase, are attached to the membrane via rhamnose-phospholipid linkage. Using a phosphostain, a study of the phosphoproteome of *Mycoplasma pneumoniae* identified 63 phosphorylated proteins. When the 2 known protein kinases of *M*. *pneumoniae* were knocked out, only 5 of the putative phosphoproteins were affected [43]. Enolase was one of 58 proteins that retained phosphostaining in mutants with disrupted protein kinase genes, suggesting that the protein was associated with phosphate indirectly by a mechanism not involving phosphorylation of amino acids, consistent with the possibility that much of the phosphoproteome of *M*. *pneumoniae* consists of proteins that lack phosphorylated amino acids and instead are attached to phospholipid.

Enolase and other glycolytic enzymes moonlight on the surface of many types of cells from bacteria to human, but the mechanism by which enolase associates with the membrane is unknown. Mammals lack rhamnose. Perhaps mammalian enolase is covalently linked to lipid by linkage with fucose or another hexose. Rhamnose is a common monosaccharide in bacteria, and walled bacteria might use rhamnose to link proteins to lipid similarly as do mycoplasmas. In general, MS facilities use reverse phase HPLC to separate peptides for analysis. The reverse phase column binds lipid tightly, and lipidated peptides would not be recovered from the column. PTMs such as those described here would likely be overlooked. We were able to recover the enolase peptide to which phospholipid was attached only after shearing. The high abundance of enolase facilitated detection.

## Supporting Information

S1 Fig MS spectrum from the peak labeled lipid in the GC/MS shown in Fig 2 panel B. This characteristic fragmentation pattern of 14 Da. increments is highly indicative of a long chain fatty acid.
(PDF)

S2 Fig MS spectrum from the GC/MS shown in Fig 2. Panel A, MS showing the characteristic ions associated with any standard methylglycoside (rhamnose, glucose, mannose, etc.). Panel B, structural representation of the ion fragments that give the 204 and 217 ions. The red circle highlights the difference between the two. Panel C, MS showing the ions associated with the rhamnose phosphate peak shown in Fig 2 panel B. The peaks at 116, 180, and 296 have been shown to be associated with sugar phosphates (Zinbo et al. 1970, manuscript reference 41). Panel D, structural representation of the 296 peak in panel C.
(PDF)

S3 Fig HR-MS spectra from the data set used to generate the plot in Fig 3. Top, spectrum from 19.74 minutes (beginning of the plot). Bottom, spectrum from 34.94 minutes (end of the plot). The similarities between the two are obvious. All intervening spectra were similar.
(PDF)

S1 Table MS/MS assignments of the peaks shown in Fig 5 for the peptide LLEIEDQLEEAAVFPGK from *M*. *pulmonis* membrane enolase. (PDF)
