# Supplementary material for: Rhamnose Links Moonlighting Proteins to Membrane Phospholipid in Mycoplasmas
Source: PLoS One. 2016 Sep 7;11(9):e0162505. doi: 10.1371/journal.pone.0162505 (PMC5014317; doi:10.1371/journal.pone.0162505)
Supplement: S1 Fig — This characteristic fragmentation pattern of 14 Da. increments is highly indicative of a long chain fatty acid. (PDF) [file pone.0162505.s001.pdf]

S1 Figure

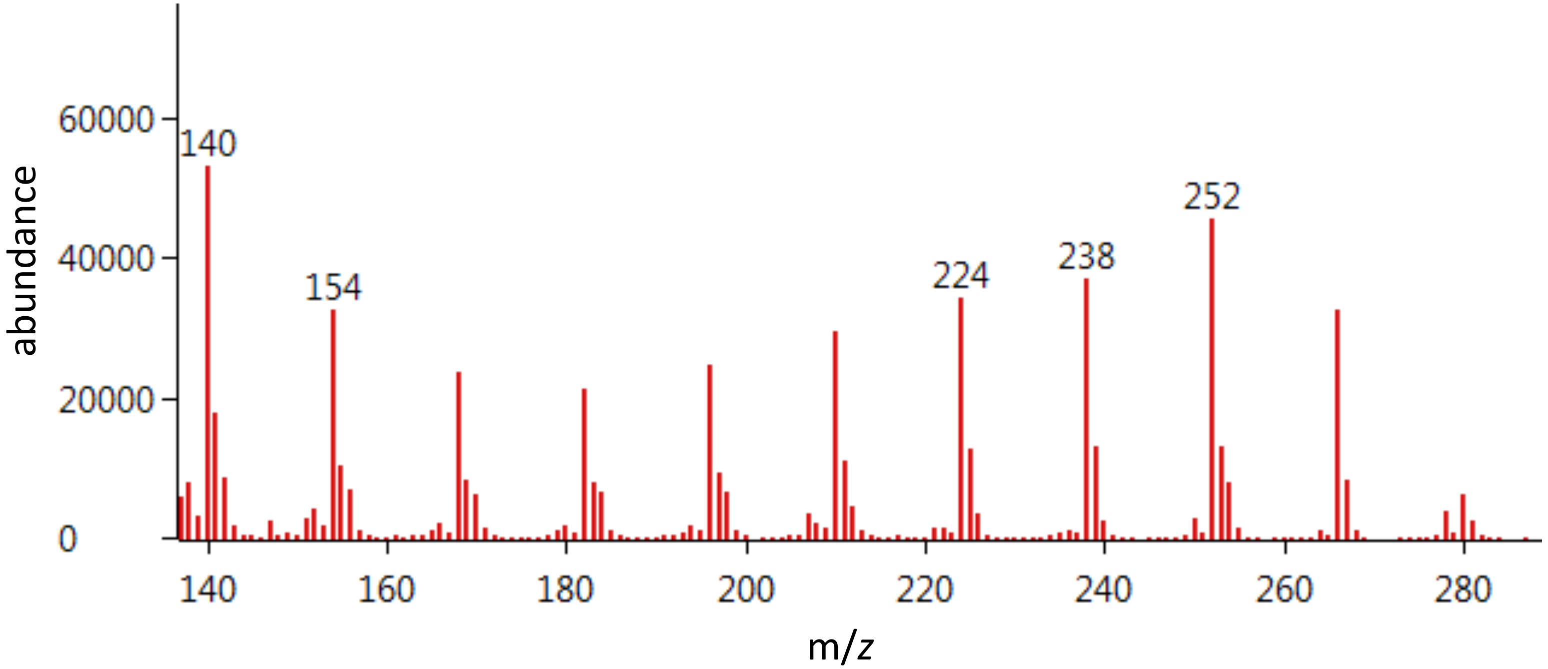

S1 Fig. MS spectrum from the peak labeled lipid in the GC/MS shown in Fig 2 panel B. This characteristic fragmentation pattern of 14 Da. increments is highly indicative of a long chain fatty acid.
