# Supplementary material for: Rhamnose Links Moonlighting Proteins to Membrane Phospholipid in Mycoplasmas
Source: PLoS One. 2016 Sep 7;11(9):e0162505. doi: 10.1371/journal.pone.0162505 (PMC5014317; doi:10.1371/journal.pone.0162505)
Supplement: S2 Fig — Panel A, MS showing the characteristic ions associated with any standard methylglycoside (rhamnose, glucose, mannose, etc.). Panel B, structural representation of the ion fragments that give the 204 and 217 ions. The red circle highlights the difference between the two. Panel C, MS showing the ions associated with the rhamnose phosphate peak shown in Fig 2 panel B. The peaks at 116, 180, and 296 have been shown to be associated with sugar phosphates (Zinbo et al. 1970, manuscript reference 41). Panel D, structural representation of the 296 peak in panel C. (PDF) [file pone.0162505.s002.pdf]

S2 Figure

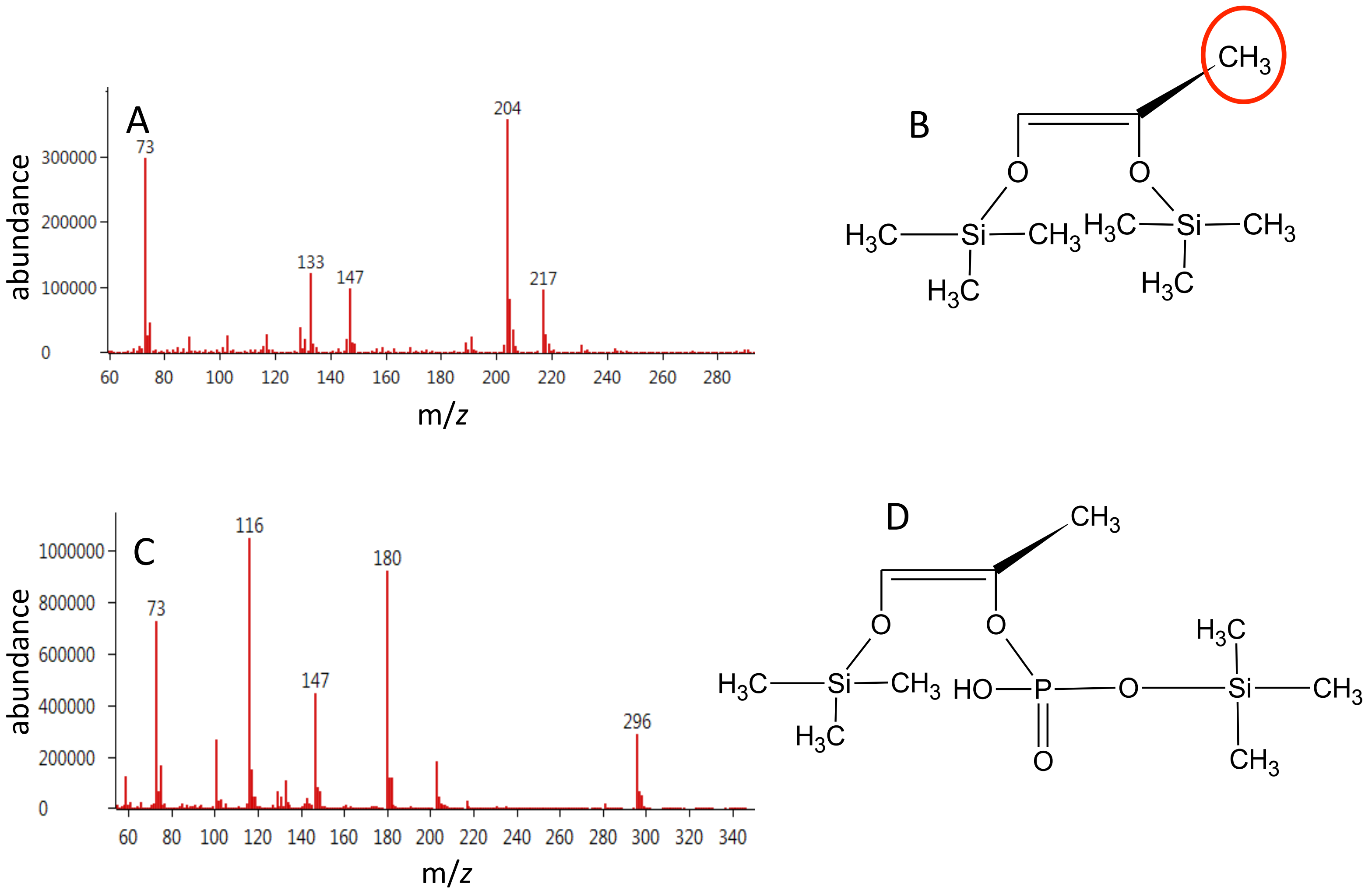

S2 Fig. MS spectrum from the GC/MS shown in Fig 2. Panel A, MS showing the characteristic ions associated with any standard methylglycoside (rhamnose, glucose, mannose, etc.). Panel B, structural representation of the ion fragments that give the 204 and 217 ions. The red circle highlights the difference between the two. Panel C, MS showing the ions associated with the rhamnose phosphate peak shown in Fig 2 panel B. The peaks at 116, 180, and 296 have been shown to be associated with sugar phosphates (Zinbo et al. 1970, manuscript reference 41). Panel D, structural representation of the 296 peak in panel C.
