# Supplementary material for: Rhamnose Links Moonlighting Proteins to Membrane Phospholipid in Mycoplasmas
Source: PLoS One. 2016 Sep 7;11(9):e0162505. doi: 10.1371/journal.pone.0162505 (PMC5014317; doi:10.1371/journal.pone.0162505)
Supplement: S3 Fig — Top, spectrum from 19.74 minutes (beginning of the plot). Bottom, spectrum from 34.94 minutes (end of the plot). The similarities between the two are obvious. All intervening spectra were similar. (PDF) [file pone.0162505.s003.pdf]

S3 Figure

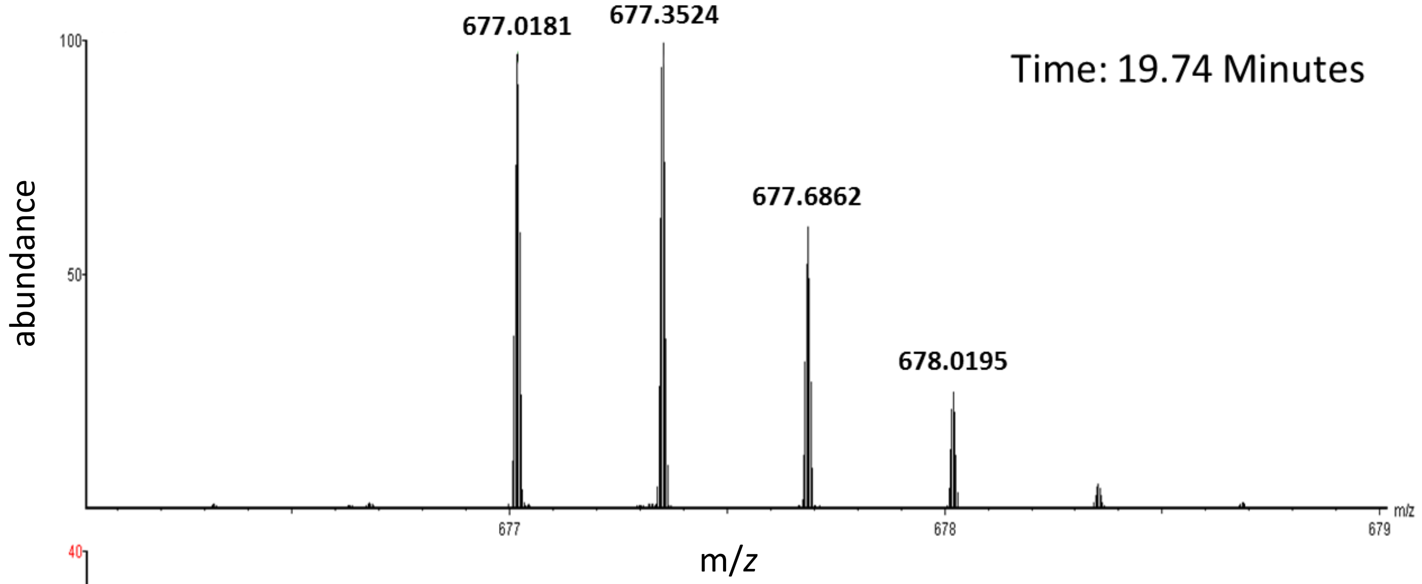

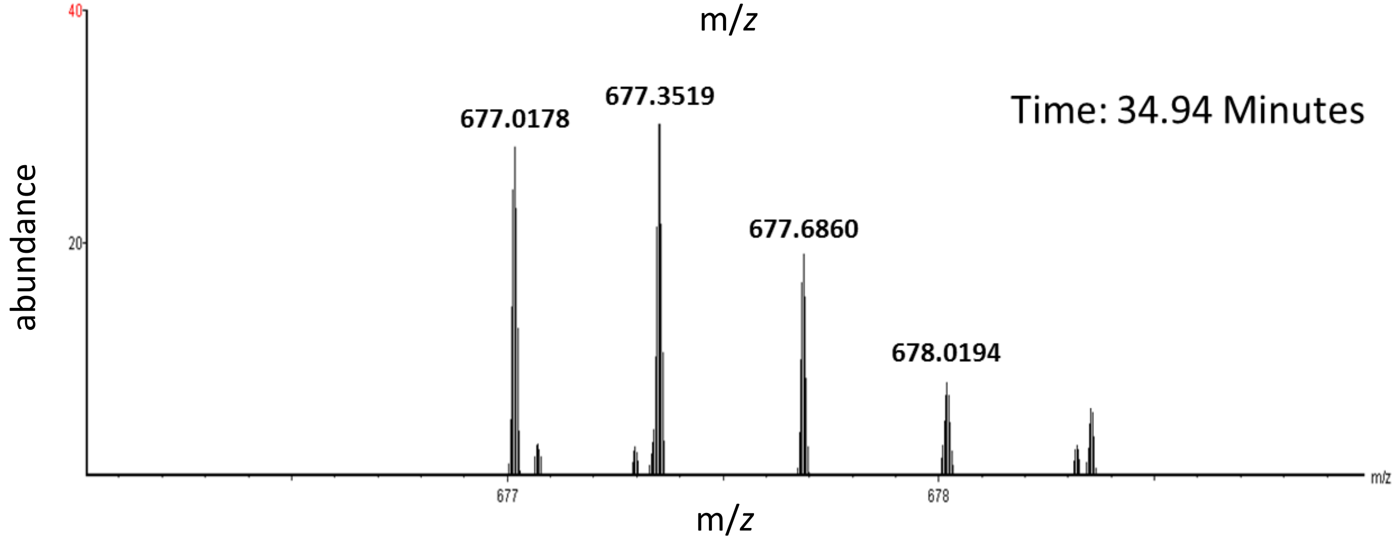

S3 Fig. HR-MS spectra from the data set used to generate the plot in Fig 3. Top, spectrum from 19.74 minutes (beginning of the plot). Bottom, spectrum from 34.94 minutes (end of the plot). The similarities between the two are obvious. All intervening spectra were similar.
