# Supplementary material for: Rhamnose Links Moonlighting Proteins to Membrane Phospholipid in Mycoplasmas
Source: PLoS One. 2016 Sep 7;11(9):e0162505. doi: 10.1371/journal.pone.0162505 (PMC5014317; doi:10.1371/journal.pone.0162505)
Supplement: S1 Table — (PDF) [file pone.0162505.s004.pdf]

Table S1. MS/MS peak assignments for the peptide LLEIEDQLEEAAVFPGK from *M. pulmonis* membrane enolase.

m/z	assignment	*m/z*	assignment
2227.08	b_2	283.08	y_3-H_2O
338.17	b_3-H_2O	301.17	y_3
356.17	b_3	430.25	y_4-H_2O
469.33	b_4	448.25	y_4
598.34	b_5	547.34	y_5
713.34	b_6	601.67	y_6-NH_3
969.51	b_7	618.34	y_6
1082.57	b_8	689.25	y_7
1211.50	b_9	818.34	y_8
1323.34	b_{10}-NH_3	947.34	y_9
1340.84	b_{10}	1060.46	y_{10}
1393.50	b_{11}-H_2O	1298.48	y_{11}-H_2O
1411.50	b_{11}	1431.67	y_{12}
1482.67	b_{12}	1542.63	y_{13}-H_2O
1581.67	b_{13}	1560.67	y_{13}
1710.50	b_{14}-H_2O	1656.17	y_{14}-H_2O
1728.83	b_{14}	1673.83	y_{14}
1825.67	b_{15}	1784.80	y_{15}-H_2O
1882.84	b_{16}	1802.84	y_{15}
		1915.84	y_{16}
